# Cartilage Quality (dGEMRIC Index) Following Knee Joint Distraction or High Tibial Osteotomy

**DOI:** 10.1177/1947603518777578

**Published:** 2018-06-02

**Authors:** Nick J. Besselink, Koen L. Vincken, L. Wilbert Bartels, Ronald J. van Heerwaarden, Arno N. Concepcion, Anne C. A. Marijnissen, Sander Spruijt, Roel J. H. Custers, Jan-Ton A. D. van der Woude, Karen Wiegant, Paco M. J. Welsing, Simon C. Mastbergen, Floris P. J. G. Lafeber

**Affiliations:** 1 Rheumatology & Clinical Immunology, UMC Utrecht, Utrecht, The Netherlands; 2 Image Sciences Institute, UMC Utrecht, Utrecht, The Netherlands; 3 ViaSana, Mill, The Netherlands; 4 Sint Maartenskliniek, Woerden, The Netherlands; 5 Department of Orthopaedic Surgery, UMC Utrecht, Utrecht, The Netherlands; 6 IJsselland Ziekenhuis, Capelle aan den IJssel, The Netherlands; 7 Department of Orthopedics, Haaglanden Medical Centre, Den Haag, Zuid-Holland, The Netherlands

**Keywords:** knee osteoarthritis, dGEMRIC, knee joint distraction, high tibial osteotomy

## Abstract

**Objective:**

High tibial osteotomy (HTO) and knee joint distraction (KJD) are treatments to unload the osteoarthritic (OA) joint with proven success in postponing a total knee arthroplasty (TKA). While both treatments demonstrate joint repair, there is limited information about the quality of the regenerated tissue. Therefore, the change in quality of the repaired cartilaginous tissue after KJD and HTO was studied using delayed gadolinium-enhanced magnetic resonance imaging of cartilage (dGEMRIC).

**Design:**

Forty patients (20 KJD and 20 HTO), treated for medial tibiofemoral OA, were included in this study. Radiographic outcomes, clinical characteristics, and cartilage quality were evaluated at baseline, and at 1- and 2-year follow-up.

**Results:**

Two years after KJD treatment, clear clinical improvement was observed. Moreover, a statistically significant increased medial (Δ 0.99 mm), minimal (Δ 1.04 mm), and mean (Δ 0.68 mm) radiographic joint space width (JSW) was demonstrated. Likewise, medial (Δ 1.03 mm), minimal (Δ 0.72 mm), and mean (Δ 0.46 mm) JSW were statistically significantly increased on radiographs after HTO. There was on average no statistically significant change in dGEMRIC indices over two years and no difference between treatments. Yet there seemed to be a clinically relevant, positive relation between increase in cartilage quality and patients’ experienced clinical benefit.

**Conclusions:**

Treatment of knee OA by either HTO or KJD leads to clinical benefit, and an increase in cartilage thickness on weightbearing radiographs for over 2 years posttreatment. This cartilaginous tissue was on average not different from baseline, as determined by dGEMRIC, whereas changes in quality at the individual level correlated with clinical benefit.

## Introduction

Knee osteoarthritis (OA) is a major socioeconomic burden.^1,2^ End-stage knee OA is most often treated with a total knee arthroplasty (TKA).^3^ When TKA is performed in patients younger than 65 years, the chance for revision surgery is significant.^4^ Revision surgery is considerably more difficult, costly, and generally less effective, leading to increased complication and mortality rates.^4^

In a population with increasing obesity, a relative younger population is increasingly at risk for development of OA. Moreover, life expectancy is increasing, increasing the risk for revision surgery later in life. Therefore, the need arises for joint preserving strategies.^5^ Since structural tissue damage is a probable cause for pain and functional limitation, joint preserving treatment focusses on tissue repair, accompanied by clinical benefit.

High tibial osteotomy (HTO) is a well-known joint preserving procedure to treat unicompartmental knee OA by correcting a deviated mechanical leg-axis, with that unloading the damaged compartment.^6-8^ Many studies show good clinical results, with high and prolonged survival rates,^6^ and even structural cartilage repair.^9,10^ A systematic review shows osteotomies can delay TKA with a median of 7 years.^11^

Knee joint distraction (KJD) is a less known joint preserving treatment and indicated for both unilateral and generalized knee OA. KJD is performed by placing an external fixation device for 6 weeks, allowing for a renewal of the joint homeostasis, where anabolic activity takes over catabolic activity, providing a more healthy environment enabling tissue repair.^12,13^ Studies have demonstrated a progressive decrease in pain, normalization of function, and a sustained increase in cartilage thickness as seen on weightbearing radiographs.^14-17^ Arthroscopy^14-16^ and magnetic resonance imaging (MRI)^14,18^ evaluation showed cartilage repair after KJD. As a surrogate marker for cartilage quality, biochemical markers for collagen type-II turnover demonstrated an increase of synthesis over release.^18^ In a prospective open uncontrolled study, KJD proved to be successful in postponing TKA for at least 5 years in more than 75% of the treated patients.^19^ Postponing a TKA over 10 years was reported to occur in two-third of patients treated with KJD based on data of small groups.^20^

HTO and KJD aim to permanently partially (HTO) or temporarily completely (KJD) alleviate the biomechanical load on the affected cartilage. Moreover, both treatments result in cartilaginous tissue repair and clinical benefit. Therefore, the effects of these treatments were directly compared in a randomized controlled trial (RCT). Recently, the 1-year evaluation of this RCT was reported.^21^ All patient-reported outcome measures were improved after 1 year (*P* < 0.02) as well as an increased joint space width (JSW) of the medial compartment on both KJD (0.8 ± 1.0 mm, *P* = 0.001) and HTO (0.4 ± 0.5 mm, *P* < 0.001). In the KJD group (in contrast to the HTO group), the lateral compartment also showed an increased JSW, resulting in a statistically significant increase in overall mean JSW (*P* < 0.02).^21^

Following reports of structural repair, the next step is to assess cartilage quality, preferably using noninvasive techniques. Quantitative MRI analysis, in the form of delayed gadolinium-enhanced magnetic resonance imaging of cartilage (dGEMRIC) relies on the relationship between the highly negatively charged glycosaminoglycans (GAG) and the negatively charged MRI contrast agent gadolinium, providing a measure of quality of the cartilaginous tissue, specifically with regard to GAG content.^22^ In OA, the highly negatively charged GAG are lost and when intravenously injected, the MRI contrast agent gadolinium, reaches the patients’ joints and penetrates the cartilage in an inverse proportional manner.^22^ The qualitative state of the cartilage is thereby represented as dGEMRIC indices; low dGEMRIC indices represent low GAG content, namely degenerated cartilage, and high dGEMRIC indices higher GAG content, namely more healthy cartilage.^23^

Although cartilaginous tissue repair is shown for both HTO and KJD, imaging data on cartilage quality are scarce. In case of HTO, there is only 1 case report series published and a few studies reporting on dGEMRIC changes; 6 months,^9,23^ 9 months,^24^ 12 months,^9,23^ and 24 months^9^ posttreatment in humans. Although positive results were obtained, none of these studies could confirm (statistically) significant cartilage quality changes on treatment with HTO. For KJD such data are not present.

In the present explorative study, the change in quality of the repaired cartilaginous tissue two years after KJD or after HTO treatment was investigated using dGEMRIC. In addition, it was evaluated whether these changes are related to radiographic changes and clinical outcome.

## Methods

### Patients

**Figure 1.**
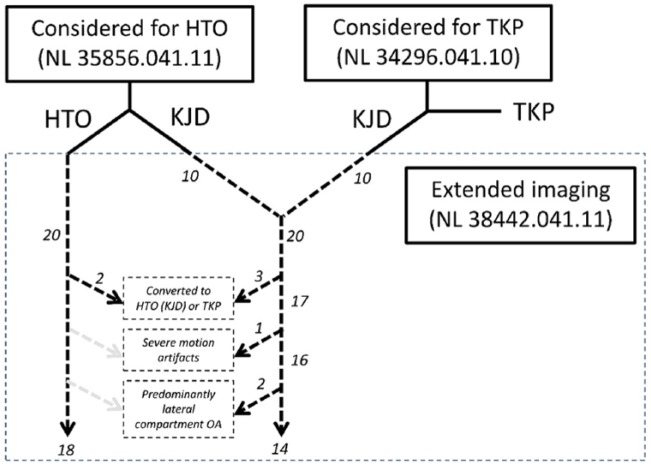
Inclusion flowchart. Patients considered for high tibial osteotomy (HTO) or total knee arthroplasty (TKP), included in either of the randomized trials (NL 35856.041.11 or NL 34296.041.10) were asked to participate in this extended imaging trial (NL 38442.041.11). Additional dGEMRIC imaging was performed at baseline and after 2 years for HTO patients, and at baseline, and after 1 and 2 years for knee joint distraction (KJD) patients.

For this explorative study patients were included originating from 2 independent RCTs (**Fig. 1**; NL 35856.041.11 and NL 34296.041.10). Patients with medial compartmental knee OA considered for HTO according to regular practice,^21^ randomized to either KJD or HTO (1:2) were asked to participate in this extended imaging study. Because of the relative low number of KJD versus HTO patients, caused by the randomization ratio, KJD patients from an RCT comparing TKA with KJD^25^ were additionally added to this study. These patients were, according to regular practice, considered for TKA surgery and randomized to either KJD or TKA (1:2).

For both studies, patients younger than 65 years, with varus deformity, Kellgren and Lawrence (K-L) score >2, intact ligaments, normal range-of-motion (flexion >120°; flexion-limitation <15°), normal stability, and a body mass index (BMI) <35 kg/m^2^ were included. Exclusion criteria included any history of inflammatory- or rheumatoid arthritis, posttraumatic fibrosis due to fracture of the tibia plateau, full bone-to-bone contact (absence of any JSW on X-ray), surgical treatment of the involved knee <6 months ago, and primary (isolated) patella-femoral OA. Patients with an infectious susceptible prosthesis in situ and/or contralateral knee OA that needed treatment were excluded as well.

After patients’ written consent to participate in 1 of the 2 RCTs, they were additionally asked to participate in the current study extending the standard MRI measurements with additional imaging modalities, including dGEMRIC to measure proteoglycan content/distribution. When comparing the demographics of the original KJD and HTO groups with those of this extended imaging study, only the proportion of males in the HTO group is statistically significantly higher, which was considered coincidental (**Table 1**).

**Table 1.** Baseline Characteristics.

		Extended Imaging Cohort			Total KJD Cohort		Total HTO Cohort	
		KJD (*n* = 14)	HTO (*n* = 18)	*P* ^a^	KJD (*n* = 42)	*P* ^b^	HTO (*n* = 45)	*P* ^b^
Age at surgery, years	Mean [95% CI]	54.14 [49.85-58.43]	48.94 [45.91-51.98]	0.044	53.14 [50.98-55.31]	0.662	49.58 [47.67-51.49]	0.715
Male	*n* (%)	9 (64)	13 (72)	0.644	25 (60)	0.215	27 (60)	0.027
BMI, kg/m^2^	Mean, [95% CI]	26.60 [24.46-28.74]	26.94 [25.52-28.36]	0.780	27.46 [26.34-28.59]	0.455	27.16 [26.18-28.15]	0.789
Left knees	*n* (%)	6 (43)	9 (50)	0.699	16 (50)	0.350	20 (44)	0.787
Kellgren and Lawrence	*n* (*n*/*N* %)	Median 3	Median 2.5	0.039	Median 3	0.486	Median 3	0.699
Grade 0		0 (0)	0 (0)		0 (0)		1 (2)	
Grade 1		1 (7)	2 (11)		6 (14)		5 (11)	
Grade 2		1 (7)	6 (39)		5 (12)		12 (27)	
Grade 3		8 (57)	8 (44)		19 (45)		23 (51)	
Grade 4		4 (29)	1 (6)		12 (29)		4 (9)	
Tibiofemoral axis	Mean [95% CI]	6.91 [4.50-9.33]	6.68 [5.33-8.03]	0.848	4.86 [3.26-6.45]	0.132	6.21 [5.53-6.89]	0.610
VAS pain	Mean [95% CI]	58.50 [45.40-71.60]	64.11 [55.79-72.43]	0.580	60.64 [53.78-67.51]	0.761	64.98 [59.47-70.49]	0.858
Baseline WOMAC	Mean [95% CI]	49.19 [40.24-58.14]	49.42 [41.75-57.10]	0.966	51.78 [46.69-56.87]	0.599	52.28 [47.13-57.44]	0.525
Baseline minimal JSW	Mean [95% CI]	0.23 [-0.16-0.62]	0.65 [0.08-1.22]	0.231	0.51 [0.22-0.80]	0.103	0.60 [0.29-0.90]	0.661
Baseline mean JSW	Mean [95% CI]	4.80 [4.31-5.30]	4.73 [4.25-5.21]	0.943	4.70 [4.36-5.04]	0.929	4.69 [4.42-4.95]	0.756
Baseline medial JSW	Mean [95% CI]	1.51 [0.53-2.49]	1.90 [1.27-2.54]	0.164	2.00 [1.32-2.69]	0.457	1.96 [1.58-2.33]	0.878
Baseline lateral JSW	Mean [95% CI]	8.61 [7.91-9.31]	7.57 [6.82-8.31]	0.044	7.40 [6.72-8.09]	0.051	7.42 [7.00-7.83]	0.610

BMI = body mass index; KJD = knee joint distraction; HTO = high tibial osteotomy; JSW = joint space width; VAS = visual analogue scale; WOMAC = Western Ontario and McMaster Universities Osteoarthritis Index.

a HTO and KJD patients’ characteristics within the extended imaging cohort are compared. As expected considering the original inclusion,^21^ age, and baseline Kellgren and Lawrence (K-L) score were statistically significant higher in the KJD group than in the HTO group. Also lateral JSW was higher for the KJD group, which was considered a coincidence. No other statistical differences were observed. For difference in K-L grade between groups, chi-square tests for trend are used. P < 0.05 is statistically significant (grayed out boxes are statistically significant).

b Demographics of the KJD and HTO patients from the extended imaging cohort are compared with their respective total cohorts.

Ethical approval was obtained (NL 38442.041.11), and the study was performed in accordance with the ethical principles from the Declaration of Helsinki. The first 20 patients who gave written informed consent treated with HTO and the first 20 patients of both RCTs treated with KJD who gave written informed consent were included.

### Treatment

**Figure 2.**
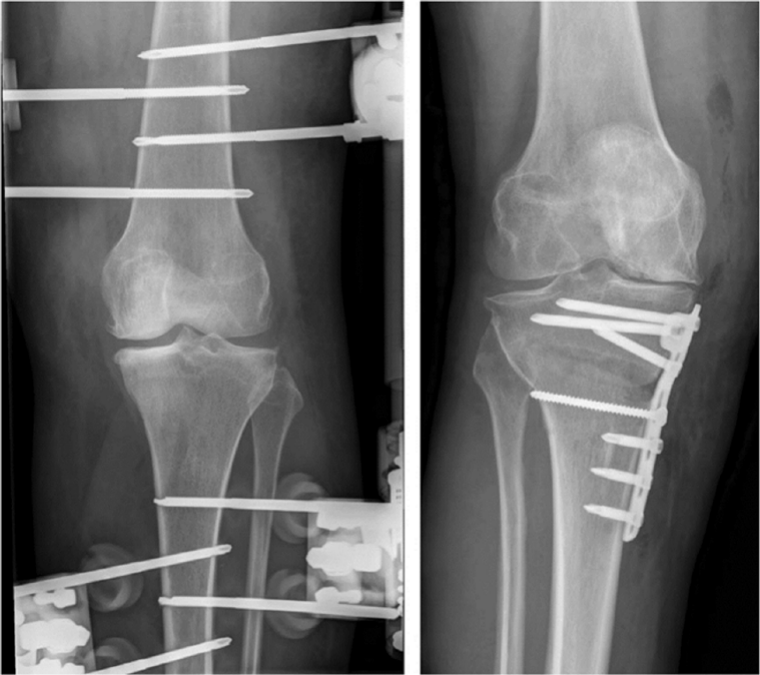
Posteroanterior radiographs of knee joint distraction (left) and high tibial osteotomy (right).

KJD was performed by placing an external fixation device, ensuring 5 mm distraction during a period of 6 weeks.^26^ In HTO, the aim was to shift the weightbearing line laterally, with the postoperative mechanical axis running laterally through the tibial plateau, at 62% of its entire width (measured from the medial side). HTO patients were hospitalized for 3 days, followed by 6 weeks of limited weightbearing. At 18 months, the plate was removed to allow MRI at 2 years. Treatment radiographs are shown in **Figure 2**. Both joint-preserving treatments have been described in more detail previously^26^ and in the supplemental file (available in the online version of the article).

### Study Assessments

For the present study, evaluations were performed before treatment (baseline), at 1 years, and at 2 years after treatment. Patients undergoing HTO did not undergo dGEMRIC MRI at 12 months due to the metal-plate *in situ*.

### Function and Pain

Clinical effectiveness was determined by the WOMAC (Western Ontario and McMaster Universities Osteoarthritis Index) 3.0 index derived from the KOOS (Knee injury and Osteoarthritis Outcome Score) questionnaire (self-assessment reduced from 5 to 3 dimensions and using a 5-point Likert-type scale, normalizing to a 100-point scale, where 100 is no pain). Pain was measured by a visual analogue scale (VAS-Pain), a continuous scale ranging from 0 (no pain) to 100 (worst imaginable pain), on which the patient indicated the amount of pain.

### Weightbearing Radiographs and Joint Space Width Measurements

Standardized semiflexed weightbearing radiographs were acquired at inclusion and 2 years after treatment to determine the K-L grade (K-L at baseline) according to a standardized protocol and to evaluate changes in JSW over time using Knee Images Digital Analysis (KIDA) software,^27^ (single experienced observer) expressed in 4 JSW measures; mean medial, and mean lateral JSW, mean of the total joint (mean JSW), and minimal JSW of the total joint. The preoperative tibiofemoral axis was measured on full leg weightbearing radiographs.

### dGEMRIC Acquisition

After scout images, dGEMRIC scans were performed on a clinical 3-tesla MRI scanner (Achieva 3T; Philips Medical Systems) using a 16-channel knee coil. The 3-dimensional imaging protocol consisted of a sagittal inversion recovery fast spoiled gradient-recalled echo (FSPGR) sequence with 5 settings for the inversion time (TI) (50; 150; 350; 650; 1650 ms), based on previously published work.^24^ An additional phantom experiment (data not shown) showed that no significant variations in measured T1 values were present over the range of slices analyzed in our study. The repetition time (TR) was 10 ms. Other parameters were: flip angle = 15°, echo time = 3 ms, field of view = 160 × 145.2 × 108 mm^3^, in-plane voxel size = 0.625 × 0.625 × 3 mm^3^, and matrix size = 260 × 234 × 36. Prior to scanning, patients received an intravenous injection of 0.2 mM/kg gadolinium-based contrast agent (Gd-DTPA; Magnevist by Bayer Schering Pharma). Subsequently, patients performed a standardized light exercise, by walking a predefined route for approximately 15 minutes, and rested until 90 minutes after contrast infusion before the MRI scan was made (dGEMRIC sequences including scout images took 20 minutes and 30 seconds).

### dGEMRIC Index Estimation

**Figure 3.**
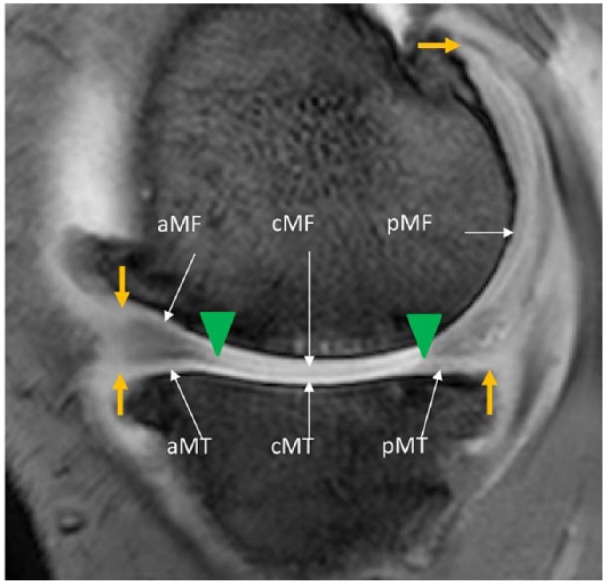
Delineating anterior (a), central (c), and posterior (p) regions of interest (ROIs) of the medial (M) and lateral (L) tibia (T) and femur (F). Regions are separated at the most anterior and posterior horn of the meniscus (green arrowheads), the anterior regions reach until the most anterior part of the tibia plateau (orange arrows). The posterior tibial region is bounded at the most posterior part of the tibia plateau, while the posterior femoral regions encompass all visible cartilage (orange arrows). Six regions are delineated per slice, for 3 consecutive slices in both the lateral and femoral compartments.

Segmentation was performed on dGEMRIC images of every patient, acquired at baseline and follow up by 2 independent observers (NB, AC), blinded for time point and treatment. This segmentation provided a total of 12 regions of interest (ROIs), divided in anterior (a), central (c) and posterior (p) regions of the tibia (T) or femur (F) on the medial (M) or lateral (L) side of the knee (**Fig. 3**). ROIs were manually delineated on the sagittal images obtained in the dGEMRIC scan with inversion time of 1650 ms (TI = 1650 ms) according to the method described by Eckstein *et al*.^24,28^ The central and both adjacent slices through both tibiofemoral joint compartments were manually selected. ROIs were delineated, using in-house developed software (ImageXplorer, Image Sciences Institute) (For interpretation of the references to colours in this figure legend, refer to the online version of this article).

Phase-corrected real data reconstruction (allowing for noise reduction), and image registration were performed on the 3-dimensional images with 5 different inversion time settings (TI = 50; 150; 350; 650; 1650 ms) before fitting.^29,30^ Eventually, all sequences were rigidly transformed to TI = 1650 ms using an intensity-based image registration, and alignment was visually inspected.

**Figure 4.**
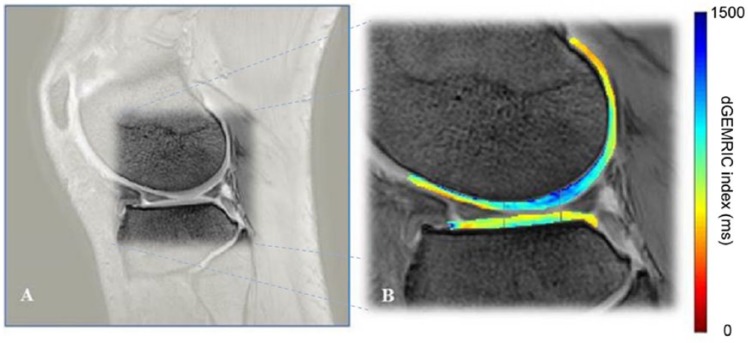
(**A**) Sagittal view of the lateral side of a tibiofemoral joint. (**B**) Automated in-house developed algorithm used to reconstruct a quantitative T1 map. The dGEMRIC index map is then superimposed onto the scan acquired for TI = 1650 ms. A color scale was used, representing the condition of the cartilage, ranging from degenerative (yellow) toward healthy (blue; low GAG content results in a low dGEMRIC index, and vice versa) (For interpretation of the references to colours in this figure legend, refer to the online version of this article).

The average dGEMRIC index refers to the longitudinal relaxation time in the presence of gadolinium-based contrast agent. Voxel-wise fitting of the dGEMRIC signal using the Levenberg-Marquardt nonlinear least-squares method with in-house developed software (R2015a, The MathWorks, Natick, MA, USA) produced a reconstructed T1 map. From this T1 map, the average dGEMRIC index was calculated for each compartment and ROI separately. The dGEMRIC index map was then superimposed onto the scan acquired for TI = 1650 ms, see **Figure 4**. A color scale was used, representing the condition of the cartilage, ranging from degenerated toward healthy (low GAG content results in a low dGEMRIC index, and vice versa).

### Statistical Analysis

Changes in WOMAC, VAS Pain, radiographic JSW, and dGEMRIC signal (per side and region) were presented using mean with SD or median with interquartile range. WOMAC, VAS Pain, and JWS changes were evaluated (without correction for multiple testing) by paired *t* tests and differences in changes scores between KJD and HTO using independent tests.

To account for clustering of dGEMRIC indices within the different regions analyzed, changes in dGEMRIC scores from baseline to follow-up, over all regions were analyzed using multilevel analysis (i.e., a linear mixed-effects model) with a random intercept at region level. In this analysis, the average change in dGEMRIC indices over time was estimated, as well as the effect of treatment and of side (medial or lateral) on this change. The association of change in dGEMRIC indices with change in WOMAC, change in JSW, and modification of these associations by side and by treatment was also evaluated with multilevel analysis and if relevant, based on size of regression coefficient of the interaction term and a *P* < 0.20, subgroup analyses were performed.

All tests were 2-sided, and a probability of *P* < 0.05 was considered statistically significant unless specified otherwise. Statistical analyses were performed using SPSS (Version 21.0. IBM Corp, Armonk, NY).

## Results

### Patients

Three out of 20 KJD and 2 out of 20 HTO patients were lost to follow-up due to conversion to HTO (in case of KJD) or total knee arthroplasty (TKA; in case of HTO) within 2 years (**Fig. 1**). In addition, 1 KJD patient had severe motion artifacts in the dGEMRIC acquisition. As the HTO patients all have medial compartment OA, 2 KJD patients with predominantly lateral compartmental OA were excluded to allow for a proper comparison between groups. This resulted in a total of 14 KJD and 18 HTO patients analyzed (see **Fig. 1**). Baseline characteristics of these patients are given in **Table 1**. There were no statistically significant differences in dGEMRIC indices at baseline between the KJD and the HTO patients.

### Clinical and Radiographic Changes after HTO or KJD

**Figure 5.**
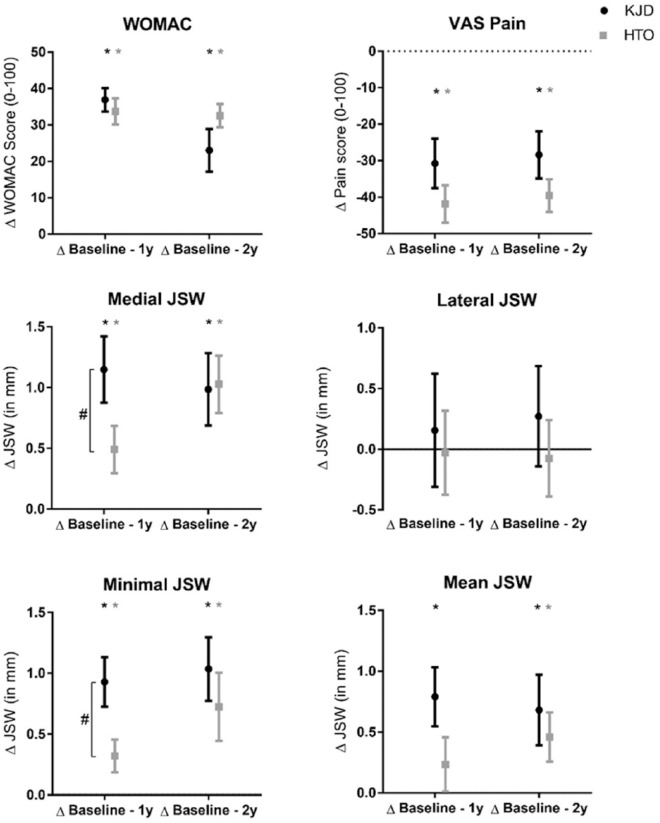
Change in WOMAC, VAS Pain, and medial/lateral/minimal/mean JSW, 1 year and 2 years after KJD or HTO. Visualized as mean change (± standard error of the mean) over 12 and 24 months, corrected for baseline. *Statistically significant (*P* < 0.05) difference over time within treatment. ^#^Statistically significant (*P* < 0.05) difference in changes over time between treatments. WOMAC = Western Ontario and McMaster Universities Osteoarthritis Index; VAS = visual analogue scale; JSW = joint space width; KJD, knee joint distraction; HTO, high tibial osteotomy.

One and 2 years after either treatment, a statistically significant increase in WOMAC and decrease in VAS-Pain compared with baseline was observed (**Fig. 5**). The 1-year results of this subcohort are fully in line with the previously published 1-year results of the entire cohorts from both original RCTs.^21,31^

One year after KJD, a statistically significant increase in medial, minimal, and mean JSW was found, this increase was still significant after 2 years. A statistically significant increase in medial and minimal JSW was found after 1 year in the HTO group, which also sustained at 2 years. After 2 years, a statistically significant increase in mean JSW after HTO was observed, which was not present at 1-year follow-up yet. JSW findings were substantiated by volumetric cartilage assessments of the delineated cartilage, total knee volume increases after both KJD and HTO, ruling out biasing of JSW changes by an altered mechanical axis (both *P* < 0.05, data not shown). Radiographic parameters did not change significantly between year 1 and 2 (**Fig. 5** and Supplemental Table 1).

There was no statistically significant difference between both treatments with regard to the change in WOMAC, VAS-Pain, and JSW parameters after 2 years. However, at 1 year after treatment, these parameters were statistically significant different for medial JSW change (KJD: Δ 1.28 mm, HTO: Δ 0.52 mm, *P* = 0.049), and minimal JSW change (KJD: Δ 0.95 mm, HTO: Δ 0.32 mm, *P* = 0.011); all in favor of KJD.

### dGEMRIC Evaluation

Interobserver reproducibility of the segmentation process was evaluated by comparing the average dGEMRIC values of the ROIs (Supplemental Figure 1). The interobserver reproducibility was high (intraclass correlation coefficient [ICC] = 0.96); therefore, dGEMRIC indices of both observers were averaged for all further analyses. Average absolute (and relative) changes in dGEMRIC values of the different medial and lateral compartments and subregions of the tibia and femur from baseline to 1-year and 2-year follow-up are shown in **Table 2** and are generally small (on average 3.4%).

**Table 2.** Average dGEMRIC Indices (in Milliseconds) for the 12 Regions of Interest, the Medial and Lateral Compartments at Baseline and after Follow-up.

		Baseline	1 Year	2 Years	Baseline – 1 Year	Baseline – 2 Years	1 Year – 2 Years
KJD	aMF	640.6 [561.5 to 719.6]	664.3 [595.4 to 733.3]	676.0 [603.3 to 748.7]	21.2 [−58.7 to 101.2]	29.9 [−47.3 to 107.2]	−11.9 [−98.3 to 74.4]
	aMT	586.6 [499.0 to 674.3]	649.7 [563.8 to 735.7]	606.6 [518.7 to 694.4]	63.1 [−45.3 to 171.5]	19.9 [−93.1 to 132.9]	−43.1 [−174.8 to 88.6]
	cMF	641.2 [571.9 to 710.5]	618.1 [546.6 to 689.5]	651.2 [581.1 to 721.4]	−15.6 [−106 to 74.8]	10.0 [−56.5 to 76.5]	18.0 [−86.7 to 122.7]
	cMT	565.4 [480.6 to 650.1]	602.2 [489.9 to 714.6]	611.6 [527.1 to 696.1]	−31.5 [−126.1 to 63.2]	41.3 [−43.8 to 126.4]	9.4 [−114.4 to 133.2]
	pMF	686.4 [635.3 to 737.4]	690.3 [638.3 to 742.2]	656.0 [603.5 to 708.5]	3.9 [−54.5 to 62.3]	−30.4 [−70.6 to 9.9]	−34.3 [−102.3 to 33.7]
	pMT	636.8 [548.0 to 725.5]	683.1 [605.2 to 760.9]	661.7 [568.4 to 755.0]	46.3 [−12.7 to 105.2]	24.9 [−56.8 to 106.7]	−21.4 [−110.6 to 67.9]
	Mean medial	640.7 [594.3 to 687.2]	653.1 [602.9 to 703.3]	642.7 [597 to 688.5]	12.4 (1.9%) [−47.6 to 72.3]	2.0 (0.3%) [−47.0 to 51.0]	−10.4 (−1.6%) [−83.9 to 63.2]
	aLF	743.6 [663.6 to 823.5]	734.8 [664.5 to 805.2]	743.3 [665.8 to 820.8]	9.9 [−78.4 to 98.3]	−0.3 [−59.1 to 58.5]	−10.4 [−110.2 to 89.4]
	aLT	699.5 [621.0 to 778.0]	724.9 [631.4 to 818.3]	731.9 [622.0 to 841.7]	25.4 [−44.3 to 95]	32.4 [−26.3 to 91.0]	7.0 [−58.1 to 72.1]
	cLF	854.7 [725.9 to 983.5]	818.5 [719.1 to 918.0]	826.2 [693.7 to 958.7]	−12.9 [−81.6 to 55.8]	−36.5 [−113.6 to 40.5]	−26.7 [−56.9 to 3.6]
	cLT	754.4 [660.7 to 848.0]	752.4 [641.6 to 863.1]	733.2 [628.2 to 838.2]	−2.0 [−84.9 to 80.9]	−21.1 [−117.9 to 75.6]	−19.1 [−86.7 to 48.4]
	pLF	789.0 [721.4 to 856.6]	785.8 [715.4 to 856.1]	780.2 [712.1 to 848.3]	−3.2 [−71.1 to 64.7]	−12.2 [−78.1 to 53.6]	3.3 [−56.7 to 63.3]
	pLT	678.0 [610.5 to 745.5]	661.7 [587.7 to 735.7]	633.3 [567.5 to 699.0]	−16.3 [−67.7 to 35.1]	−44.7 [−118.4 to 29.0]	−28.4 [−81 to 24.1]
	Mean lateral	763.4 [691.5 to 835.3]	754.4 [691.7 to 817.1]	754.4 [685.6 to 823.2]	−9.0 (−1.2%) [−62 to 44]	−9.0 (−1.2%) [−61 to 43]	0.0 (0.0%) [−40.7 to 40.7]
HTO	aMF	686.7 [599.8 to 773.6]		622.6 [554.1 to 691.0]		−64.1 [−153.3 to 25.0]	
	aMT	595.3 [525.6 to 665.0]		613.7 [550.4 to 677.0]		18.4 [−33.5 to 70.3]	
	cMF	692.4 [576.3 to 808.5]		574.6 [515.2 to 633.9]		−117.7 [−243 to 7.6]	
	cMT	594.9 [534.3 to 655.6]		651.5 [591.8 to 711.2]		48.0 [−3.4 to 99.4]	
	pMF	726.9 [667.2 to 786.7]		679.7 [626.0 to 733.3]		−45.7 [−126.1 to 34.7]	
	pMT	711.4 [654.8 to 768.1]		697.9 [641.5 to 754.2]		−13.6 [−58.0 to 30.9]	
	Mean medial	679.3 [617.4 to 741.1]		662.3 [618.1 to 706.5]		−17.0 (−1.0%) [−79.4 to 45.4]	
	aLF	778.6 [705.5 to 851.7]		726.7 [651.6 to 801.9]		−51.9 [−129.3 to 25.5]	
	aLT	775.0 [717.3 to 832.7]		785.1 [715.4 to 854.7]		10.1 [−62.1 to 82.2]	
	cLF	902.8 [813.8 to 991.7]		811.0 [725.3 to 896.7]		−90.1 [−169.4 to −10.9]	
	cLT	793.9 [720.3 to 867.6]		829.8 [778.7 to 880.9]		35.8 [−25.1 to 96.8]	
	pLF	793.9 [726.0 to 861.7]		775.3 [703.9 to 846.7]		−21.4 [−106.2 to 63.3]	
	pLT	674.0 [636.1 to 711.9]		696.8 [646.8 to 746.7]		22.8 [−31.9 to 77.5]	
	Mean lateral	787.1 [741.1 to 833.2]		772.7 [719.9 to 825.6]		−14.4 (−1.2%) [−68 to 39.2]	

a The 12 regions of interest (ROIs) are the anterior (a), central (c), and posterior (p) regions of the Lateral (L) or Medial (M) compartment of the Femur (F) and Tibia (T). Delta scores might deviate from the difference between time points due to missing dGEMRIC indices for specific ROIs at specific time points. Missing indices can, for example, be caused by cartilage being reduced to a volume so small, it is insufficient for dGEMRIC analysis.

In the multilevel analysis, the overall average dGEMRIC change over 2 years was nonsignificant (∆ −8.08; 95% CI = −24.46 to 8.29, *P* = 0.260). dGEMRIC changes were dependent on baseline dGEMRIC indices. Taking this into account a statistically significant effect for side was found and a possible effect of treatment was found. **Table 3** shows the effect of treatment on change in dGEMRIC indices (corrected for the dGEMRIC baseline indices), for subgroups regarding side and treatment type. Of both treatments, HTO was associated with a statistically significant reduction (cartilage worsening) in medial dGEMRIC indices (∆ −44.93, 95% CI = −67.94 to −21.91) and increase (cartilage improvement) at the lateral side (∆ +26.36, 95% CI = +2.71 to +50.03). For KJD, the changes over 2 years were not statistically significant (**Table 3**). Relative changes compared with baseline were minimal^32^ (HTO medial: −6.6%, *P* < 0.001 and lateral +3.3%, *P* = 0.023 and KJD medial: −3.2% and lateral +2.1%).

**Table 3.** The Effect of Joint Sparing Treatments on dGEMRIC Indices, Linear Mixed-Effects Models.^a^

Subgroup^b^	Estimate^c^	95% Confidence Interval		Significance (*P* Value)
		Lower Bound	Upper Bound	
HTO lateral	26.36	2.71	50.03	0.029
HTO medial	−44.93	−67.94	−21.91	<0.001
KJD lateral	11.65	−14.39	37.70	0.380
KJD medial	−23.07	−49.52	3.37	0.087

HTO = high tibial osteotomy; KJD = knee joint distraction.

a All models were controlled for baseline dGEMRIC indices. Grayed out boxes are statistically significant.

b dGEMRIC indices from baseline over all regions were analyzed using multilevel analysis (i.e., a linear mixed-effects model), a random intercept at the region level was included to account for clustering of dGEMRIC indices within regions. The effect treatment (KJD or HTO), side of the knee (medial and lateral) on change in dGEMRIC indices were evaluated as fixed effect in the model. Change in dGEMRIC index was statistically significantly related to side (*P* < 0.001), but not to treatment (*P* = 0.8002), but the interaction term indicated that the effect of treatment may be modified by side (*P* = 0.09). So, effects per subgroup (HTO lateral/HTO medial/KJD lateral/KJD medial) were estimated in the model.

c Mean change in dGEMRIC indices per subgroup (as a result of treatment in a knee compartment).

### Association between Change in Radiographic and Clinical Parameters and Change in dGEMRIC

Evaluating the association between change in JSW and change in dGEMRIC over 2 years, possible effect modification was also observed by side and treatment and thus results were stratified by side and treatment (**Table 4**). Only the positive association between the change in lateral JSW and change in lateral dGEMRIC indices in patients treated with HTO were observed; where one mm increase in JSW was associated with an increase of about 26 dGEMRIC ms (*P* = 0.007, **Table 4**). This effect was not found for the medial compartment and not found after KJD for either of the 2 compartments.

**Table 4.** The Association of Change in dGEMRIC Indices with Change in Joint Space Width (JSW) and Change in WOMAC Evaluated Using Linear Mixed-Effects Models.^a^

		Estimate^b^	95% Confidence Interval		Significance (*P* Value)
			Lower Bound	Upper Bound	
∆dGEMRIC vs. ∆JSW^c^	KJD medial	0.49	−23.04	24.02	0.968
	KJD lateral	0.01	−18.40	18.43	0.999
	HTO medial	−14.84	−41.39	11.70	0.276
	HTO lateral	25.73	7.49	43.96	0.007
∆dGEMRIC vs. ∆WOMAC^d^		1.59	0.67	2.51	<0.001

WOMAC = Western Ontario and McMaster Universities Osteoarthritis Index; HTO = high tibial osteotomy; KJD = knee joint distraction.

a All models were controlled for baseline dGEMRIC indices. Grayed out boxes are statistically significant.

b One unit of JSW/WOMAC change is related to this average change in dGEMRIC indices.

c A statistically significant effect for side of the knee was found (*P* < 0.001). Evaluating modification of the association between JSW change and dGEMRIC change by side in the regression model also indicated that effect modification may be present (regression coefficient: 14.62, *P* = 0.20), thus all further analyses were stratified by side. Hereafter, modification of the association between JSW change with dGEMRIC change by treatment was evaluated (regression coefficient of −30.57, *P* = 0.03), justifying additional stratification by treatment.

d A statistically significant effect for side of the knee (*P* < 0.001) and treatment (*P* < 0.001) was found. Evidence for modification of the association between change in WOMAC and dGEMRIC change by side or by treatment was not found (WOMAC * side: *P* = 0.71, and WOMAC * treatment: *P* = 0.42), thus the group was not stratified for treatment and/or side.

For the association between change in WOMAC and change in dGEMRIC over 2 years, no evidence for modification of the association by side or by treatment was found and thus results were applicable to the total group (KJD and HTO). Results indicate that one unit increase in WOMAC (clinical improvement) was associated with an increase (tissue structure improvement) in dGEMRIC indices of about 1.6 ms (*P* < 0.0001, **Table 4**).

## Discussion

In these subcohorts, clear clinical improvement and radiographic cartilaginous tissue repair were found, without significant change in cartilage quality as determined by dGEMRIC at 2 years after KJD or HTO treatment. An increase in dGEMRIC signal, increase in cartilage GAG content, namely quality improvement, seems to correlate with an increase in clinical benefit as determined by WOMAC.

For this study, patients were included originating from 2 separate RCTs. There were differences in baseline characteristics (of inclusion criteria) for those 2 RCTs, which was reflected in the extended imaging cohort where a higher age and a more severe K-L grade for KJD at baseline as compared with HTO was found. This can be explained by the fact that part of the included KJD patients (10 out of 20) were originally considered for TKA, and these patients generally suffer from more severe OA than patients considered for HTO. The current study might be underpowered to provide final conclusive answers due to the relative low numbers of patients included. Despite these limitations, these are the first data on comparing cartilage quality between these regenerative treatments.

One of the main reasons for patients to undergo treatment of an OA knee is to alleviate pain and recover function. Even in this small study, both are achieved as seen in the clear decrease in VAS-Pain and increase in WOMAC scores, 1 year after treatment and maintained for another year, after either KJD or HTO. Interestingly, despite minor changes in dGEMRIC signal, for the overall group, change in WOMAC score was positively associated with a change in dGEMRIC indices, independent of side or of treatment, implying a clinically relevant correlation between increase in cartilage quality as determined by dGEMRIC and patients’ experienced clinical benefit. The mechanism behind this interrelation can only be speculated on.

After correction for baseline dGEMRIC indices over all ROIs, no statistically significant differences between HTO and KJD on change in dGEMRIC values were found. On average, there is a decrease in medial and an increase in lateral dGEMRIC indices for HTO patients. This increase in GAG content at the lateral compartment after HTO and decrease at the medial compartment might be the result of wedging of the joint after HTO, resulting in a slight lateral compression and a slight medial decompression, and with that relative (apparent) change in GAG signal. This is supported by a study demonstrating the sensitivity of dGEMRIC values to cartilage compression and unloading.^33^ Change in dGEMRIC indices are, on average, all quite small, representing relative small changes in cartilage quality over 2 years. The assumption of compression of the lateral compartment is however not supported by the observation that a significant relation between a decrease in lateral JSW and a decrease in cartilage quality (dGEMRIC indices) was found in specifically the lateral compartment of HTO patients. This positive association between change in JSW and change in dGEMRIC signal in specifically the lateral compartment indicates that in case of an increasing lateral joint space width, despite wedging of the whole joint, quality of cartilage (higher dGEMRIC score) improves in these cases, over 2 years. So, this might represent actual improvement of quality accompanying an increase in JSW. However, the fact that this is only found in the lateral compartment on only HTO treatment and that absolute changes are small argues its relevance.

No statistically significant relation between structural change and dGEMRIC change in KJD patients was found. dGEMRIC values are expected to improve only if cartilage damage is at the early stage, whereas if the collagen structure is already compromised, a replenishment of GAGs becomes more difficult, which could explain the statistically significant influence of baseline dGEMRIC values on the change over time. The lack of statistically significant or consistent change in dGEMRIC values for KJD, together with the clear increase in JSW, suggests that the tissue quality in KJD patients, on average, including the newly formed, is maintained. It might be argued whether this quality is sufficient, as baseline values are obtained from presumably impaired cartilage tissue in a severely damaged OA joint. Unfortunately, the dGEMRIC signal of the baseline condition of the treated joints was not compared with the contralateral healthy joint. Since dGEMRIC values are expected to decrease over time in damaged joints, although no data are available, the maintenance of cartilage quality over time could be considered a positive finding. KJD and HTO may have been useful in stopping further cartilage degeneration, indicated by minor or absent changes in dGEMRIC indices. The question remains whether there is an increase in cartilage quality of the residual tissue with newly formed tissue of inferior quality, whether the new tissue is of similar quality as the residual unchanged tissue, or whether it is residual cartilage tissue that has decompressed and thereby showed an apparent decrease in quality (lower GAG content per volume).

It was subjectively observed that cartilage quality in the deeper layers (on to the bone) seemed to improve over two years (representative image shown in Supplemental Figure 2). In the original MRI KJD studies, it was demonstrated that newly formed tissue is largely filling up denuded bone areas, thus cartilaginous tissue is formed in the deep layers.^34^ This is suggestive of newly formed quality tissue, filling in denuded bone area’s but is far from conclusive.

With regard to the dGEMRIC imaging technique; a series of scans, acquired with different echo times, is necessary to calculate dGEMRIC indices. Increased scanning times increase the risk of patient motion in between sequences (repositioning), potentially decreasing the efficacy of the fitting. Repositioning effects in our study were minimized by implementing image registration.^35^ Longitudinal evaluation of cartilage repair, such as represented in this explorative study, assume equal distribution of gadolinium within the joint. Although our contrast protocol is very strict, variations are inevitable, amongst others because of heterogeneous uptake of gadolinium in repair tissue over time, influenced not only by GAG content but also patient motion, water content, and permeability of tissue.^36,37^ Note that it takes also quite some time for the contrast to distribute throughout the body. This variation may add to the inability to detect small changes over time.

GAG concentration is, given its substantial contribution to load-bearing, a good measure to distinguish healthy from degenerated tissue.^22^ However, studies have shown that some results cannot be explained by GAG measurements alone, but might be found in a combination of several quantitative MRI techniques, morphological, and clinical evaluation.^22,38^ dGEMRIC is considered a valuable tool in evaluating cartilage quality, but there are also alternative MRI techniques available to assess cartilage quality, such as sodium MRI, T1 rho, and T2-mapping.^22^

All limitations of dGEMRIC imaging considered in general and in this specific small size study, implementation of a strict contrast administration protocol, minimized patient motion during acquisition, postprocessing image registration, and minimal variation between observers should be sufficient to consider dGEMRIC indices as representative for cartilage quality with respect to GAG content/distribution in this study. Assuming this, despite the limited number of patients, it might be concluded that cartilaginous repair on HTO and KJD is not accompanied by further decrease in GAG content. Future studies powered to elucidate potential differences between HTO and KJD treatment on dGEMRIC indices should be performed to support current findings and provide conclusive answers.

Summarizing, the significant clinical benefit and increase in radiographic JSW 1 year after treatment of medial compartmental OA by either HTO or KJD, maintains throughout the second year of follow-up, postponing the natural OA progression rate and with that knee arthroplasty. There seems to be a clinically relevant relation between the increase in cartilage quality as determined by dGEMRIC and patients’ experienced clinical benefit determined by WOMAC. Assuming natural deterioration of the cartilage tissue seen in osteoarthritis patients, is reflected in loss of GAG and therefore also applies to a decrease in dGEMRIC indices, KJD and HTO may contribute to regeneration of cartilaginous tissue with maintenance of cartilage quality, and thereby delaying the degeneration process.

## Supplementary Material

Supplementary Material, Supplementary_file_cartilage_02-02-2018 – Cartilage Quality (dGEMRIC Index) Following Knee Joint Distraction or High Tibial Osteotomy Click here for additional data file. Supplementary Material, Supplementary_file_cartilage_02-02-2018 for Cartilage Quality (dGEMRIC Index) Following Knee Joint Distraction or High Tibial Osteotomy by Nick J. Besselink, Koen L. Vincken, L. Wilbert Bartels, Ronald J. van Heerwaarden, Arno N. Concepcion, Anne C. A. Marijnissen, Sander Spruijt, Roel J. H. Custers, Jan-Ton A. D. van der Woude, Karen Wiegant, Paco M. J. Welsing, Simon C. Mastbergen, and Floris P. J. G. Lafeber in CARTILAGE
