# Supplementary material for: Cartilage Quality (dGEMRIC Index) Following Knee Joint Distraction or High Tibial Osteotomy
Source: Cartilage. 2018 Jun 2;11(1):19–31. doi: 10.1177/1947603518777578 (PMC6918034; doi:10.1177/1947603518777578)
Supplement: Supplementary Material, Supplementary_file_cartilage_02-02-2018 – Cartilage Quality (dGEMRIC Index) Following Knee Joint Distraction or High Tibial Osteotomy [file Supplementary_file_cartilage_02-02-2018.pdf]

Supplementary information: Treatments

Knee joint distraction

Knee joint distraction was performed by use of an external distraction device. On both the medial and lateral side of the femur and the tibia, two half-pins were placed, to be attached to an external fixation device (see figure 2). Intra-operatively 2 mm distraction was applied, and post-operatively 1 mm per day until 5 mm was reached. At day four a weight-bearing radiograph was taken, ensuring 5 mm distraction was applied with respect to the pre-operative weight-bearing radiograph. The patient was then discharged and full weight-bearing was encouraged. Three weeks after the operation, the pintracts were checked for infections and distraction distance was assessed on weight-bearing radiographs. Six weeks after the operation the distraction distance was checked for the last time prior to removal of the frame under general/spinal anesthesia. While under anesthesia, the knee was brought into flexion, stretching the fibrotic scar tissue around the pintracts. Patients were advised to gradually increase weight-bearing of the joint with 10 to 15 kg per week and physiotherapy on demand. Prophylactic low molecular weight heparine was given for nine weeks, during the distraction period and the three weeks thereafter.

High tibial osteotomy (medial opening wedge)

Medial opening wedge osteotomy aims at offloading the affected medial compartment of the knee by displacing the mechanical axis to the unaffected lateral compartment of the knee (from varus to valgus). Using weight-bearing radiographs, the method of Miniaci was utilized to determine the optimal degree of correction [19]. When performing the osteotomy the lateral cortex was left intact and the tibia was fixed with a plate. Patients were hospitalized for a maximum of three days before being discharged, followed by six weeks of limited (max 15 kg), weight-bearing. Knee flexion and extension was unrestricted. Prophylactic low molecular weight heparin was given until six weeks after the HTO. Six weeks after the HTO, stability was evaluated, and radiographs were taken to assess consolidation, if deemed sufficient, full weight-bearing was allowed and physiotherapy was recommended. At eighteen months the plate was removed to enable MR imaging.

Supplementary information: Changes over time

Table S1. Changes in WOMAC, VAS Pain, and JSW between baseline, one year follow-up and two-year follow-up. Double-sided T-tests, adjusted for multiple testing with false discovery rate (FDR) = 1%. * P < 0.05 is statistically significant. Greyed out boxes are statistically significant. [#]Joint space width values are corrected for baseline.

			Baseline	*1 year*	*2 year*	*Baseline – 1y*	*P**	*Baseline - 2y*	*P**	*1y – 2y*	*P**
KJD	*WOMAC (0-100)*		49,19 [40,24 to 58,14]	86,08 [80,67 to 91,49]	73,61 [60,42 to 86,80]	36,89 [29,90 to 43,88]	0,000	23,04 [10,19 to 35,89]	0,002	-12,08 [-23,31 to -0,84	0,037
KJD	*VAS (0-100)*		58,50 [45,40 to 71,60]	27,71 [15,38 to 40,04]	28,00 [11,59 to 44,41]	-30,79 [-45,48 to -16,09]	0,000	-28,38 [-42,51 to -14,26]	0,000	-1,85 [-11,84 to 8,14]	0,976
KJD	*JSW[#]*	*Medial (in mm)*	0,00	1,14 [0,59 to 1,69]	1,06 [0,44 to 1,68]	1,15 [0,56 to 1,74]	0,000	0,99 [0,33 to 1,64]	0,002	-0,18 [-0,65 to 0,28]	0,690
KJD	*JSW[#]*	*Lateral (in mm)*	0,00	0,21 [-0,73 to 1,15]	0,40 [-0,47 to 1,27]	0,16 [-0,85 to 1,16]	0,741	0,27 [-0,63 to 1,17]	0,500	0,25 [-0,63 to 1,13]	0,854
KJD	*JSW[#]*	*Minimal (in mm)*	0,00	0,93 [0,53 to 1,34]	1,11 [0,56 to 1,66]	0,93 [0,49 to 1,37]	0,000	1,04 [0,46 to 1,61]	0,000	-0,01 [-0,68 to 0,65]	0,749
KJD	*JSW[#]*	*Mean (in mm)*	0,00	0,79 [0,26 to 1,32]	0,68 [0,05 to 1,31]	0,79 [0,26 to 1,32]	0,003	0,68 [0,05 to 1,31]	0,027	-0,11 [-0,65 to 0,43]	0,774
HTO	*WOMAC (0-100)*		49,42 [41,75 to 57,10]	83,16 [73,75 to 92,57]	81,94 [73,16-90,73]	33,74 [26,13 to 41,34]	0,000	32,52 [25,79 to 39,25]	0,002	-1,22 [-5,93 to 3,50]	0,843
HTO	*VAS (0-100)*		64,11 [55,79 to 72,43	22,22 [9,720 to 34,72]	29,47 [17,54 to 41,40]	-41,89 [-52,76 to -31,02]	0,000	-39,56 [-48,97 to -30,14]	0,000	4,00 [-7,10 to 15,10]	0,265
HTO	*JSW[#]*	*Medial (in mm)*	0,00	0,49 [0,08 to 0,91]	1,03 [0,53 to 1,53]	0,49 [0,08 to 0,91]	0,017	1,03 [0,53 to 1,53]	0,000	0,54 [0,12 to 0,95]	0,089
HTO	*JSW[#]*	*Lateral (in mm)*	0,00	-0,03 [-0,76 to 0,71]	-0,07 [-0,74 to 0,59]	-0,03 [-0,76 to 0,71]	0,936	-0,07 [-0,74 to 0,59]	0,814	-0,05 [-0,84 to 0,75]	0,922
HTO	*JSW[#]*	*Minimal (in mm)*	0,00	0,32 [0,04 to 0,60]	0,72 [0,13 to 1,32]	0,32 [0,04 to 0,60]	0,023	0,72 [0,13 to 1,32]	0,015	0,40 [-0,14 to 0,95]	0,202
HTO	*JSW[#]*	*Mean (in mm)*	0,00	0,23 [-0,24 to 0,71]	0,46 [0,03 to 0,89]	0,23 [-0,24 to 0,71]	0,302	0,46 [0,03 to 0,89]	0,030	0,23 [-0,20 to 0,65]	0,459
